# Microbial pathogens in the movies

**DOI:** 10.1093/femsle/fnad129

**Published:** 2023-12-06

**Authors:** Manuel Sánchez-Angulo

**Affiliations:** Department of Vegetal Production and Microbiology, Universidad Miguel Hernández, Elche, Alicante 03202, Spain; Alicante Institute for Health and Biomedical Research (ISABIAL), Hospital General Universitario Dr. Balmis. Avda. Pintor Baeza, Nº12 Edificio Gris, 5ª planta - Alicante 03010, Spain

**Keywords:** education, film, popular culture, science communication

## Abstract

Usually, show business depicts viruses, bacteria, and other microorganisms as one of the worse menaces to mankind. Entertainment movies influence the way audiences understand and perceive these topics. Few films accurately portray the science of microbiology and its social implications. Movies and TV series often feature outbreaks of deadly diseases and the efforts of scientists and medical professionals to contain them. However, entertainment movies can also be used to educate the public about the importance and the impact that microorganisms have on our lives, helping to increase public awareness and appreciation of the world of microbiology. The aim of this review is to show the relationship between movies and microbiology, from the fight against diseases such as AIDS or tuberculosis, to the zombie apocalypse.

## Teaser

Review of scientific and cultural aspects of microbiology in film and television and the use in education.

## Introduction

Maybe one day the Academy of Motion Picture Arts and Sciences will honor an Oscar award for microbes. There are numerous films in which they have participated [according to the Internet Movie Database (IMDB), as of 12 June 2023, there are 22 502 titles related to the keyword “virus,” 184 related to “bacteria,” and 760 to “infection”). To give an example: without “viruses” we would not have such cinematic characters as the zombies that try to eat Brad Pitt and his family in *World War Z*, or the cool vampires from the *Blade* saga. Probably microbes will never have their well-deserved prize because of their dreadful reputation. In entertainment movies, microbes usually play the “bad bugs” role, like the ones that cause plague, cholera, Ebola, AIDS, or zombie outbreaks (Pappas et al. 2003, Burns and Bhella 2017). Most of the time their task is reduced to being the cause of a terrible disease that puts the protagonists in trouble or even kills them.

Although in nature, microbes do much more good than harm, this aspect is rarely represented on the screen. Something logical if we think that cinema is an art and as such appeals to the emotions, so the depiction of the suffering of an infectious disease arouses many more feelings than the operation of a bioreactor for penicillin production. We must not forget that a movie is an artistic recreation of reality, not the real world. With the exception of documentary cinema, commercial movies do not need to be scientifically precise. Nevertheless, some film productions, specially the most recent ones, take great care in enrolling scientific advisors in order to have the most plausible and realistic scripts (Chambers 2017). However, the movies fix in the public’s imagination a certain cliché of the concepts and events that are recreated on film. If we consider that our students are part of that public, it is not rare to use some famous movie sequences as an educational tool (Rose 2003, Sánchez 2011a, Baños and Bosch 2015, Berlin 2016, Schneider 2016). Of course, any educator likes to see that the subject he teaches is reflected with a certain accuracy on the big screen and that there are few “artistic licenses.” However in most occasions that situation does not occur, so making a virtue out of necessity, movie goofs such as the scene from *Mission: Impossible II* where a flu virus infects a red blood cell, can be seen as an opportunity to explain the concept and conditions of viral host recognition to the students.

The aim of this work is to give an overview of the fascinating facets of the pathogenic microorganisms depicted in entertainment movies and its use as an educational tool for the explanation of several concepts—scientific, social, and historical—at different educational levels, from undergraduate to grade students or even general public. Nowadays, the internet, video sharing platforms, and the different streaming services provide easy access to a wide range of movies and TV series, no matter whether they are old silent black-and-white films, timeless classics, modern blockbusters, or even forgotten flops. So, educators are not limited to relying solely on the most well known or current movies to illustrate specific concepts related to infectious diseases. However, we must not forget that, when a movie is made, it typically reflects the issues and concerns of the period when it was made, not the period that is reenacted on the screen (e.g. Bergman’s masterpiece, *The Seventh Seal*, is more about the experiences of life, such as love, death, and family, rather than painting a faithful representation of the Middle Ages in Scandinavia). Movies can be used in the classroom in two ways depending on the time available. The students can watch the whole film as an external activity and then discuss its content in the class (Baños and Bosch 2015). Another option is to use clips of selected scenes related to the subject to be discussed in a manner similar to the “microbe minute” strategy (Feldman 2013). Let us see a couple of examples that have been used in educational activities. At minute 19 of *Dallas Buyers Club*, a medical doctor (Jennifer Garner) explains to her AIDS patient (Matthew McConaughey) what is a doubled-blind clinical trial, which can be used as introduction to a class about bioethics and/or drug development. Another example is the sequence at minute 74 from the movie *Panic in the streets*, which can be used to explain the “One Health” concept (Sanchez 2022), thanks to the following dialogue:

Community? What community? Do you think you’re living in the Middle Ages? Anybody that leaves here can be in any city in the country within 10 hours. I could leave here today and be in Africa tomorrow. And whatever disease I had would go with me… Then think of it when you're talking about communities! We're all in a community…the same one!

It is important that the educator must watch the film beforehand and prepare the session to reach the educational objectives, for example making a questionnaire about the pathogen or the treatment portrayed on the screen, or the errors and misconceptions found. In the first example mentioned above, the character played by McConaughey believes in the effectiveness of quackery treatments for AIDS, so a good exercise would be to ask the students what the appropriate treatments would be. These clips can also be used in popular science activities aimed at the general public. One of the best clips for that purpose is from the animated short film *Your Friend the Rat*. In <3 min, it describes how the bacteria *Yersinia pestis* caused the 1347 bubonic plague pandemic known as “The Black Death” thanks to the role as a vector of the flea (*Xenopsylla cheopis*) that transmitted the bacteria to the black rat (*Rattus rattus*) causing its death, so the flea must search for another host, the humans.

This review has been organized in three major sections. The first one is dedicated to the real infectious diseases that have been represented on the screen. Commercial movies are better known to the general public than documentaries, so they could be used to explain the symptoms of the illness but also other humanistic aspects such as the care of the sick or the impact of the disease in a particular historical moment or in the actual society. The second section is dedicated to the movies where the protagonists are the scientists and medical doctors who fight against those diseases. In this case, the main concepts to be explained are the scientific methods and the biocontainment measures, but also the evolution of the stereotype of the scientist, from the solitary, dedicated hero of the 1930s to today’s interdependent groups of highly technical researchers. Finally, the last part deals with the films in which microbes manage to defeat humanity, creating a dystopian world. Those movies are well known by the general public and can be used to explain key concepts in epidemiology and disease transmission but also how to deal with extreme stressful situations (Kendal 2021).

## Diseases

Gregorio Marañón stated that illness is a part of “the dramatic side of life,” and in fact the representation of illnesses is constant in the history of arts. Probably, the first pictorial illustration of an infectious disease is the famous Egyptian stele from the 18th Dynasty that represents a person with polio (Front de Mora 2015). Bocaccio’s *Decameron* was conceived after the plague epidemic of 1348. The Black Death was also the inspiration of the allegorical Dance of Death, or *Danse Macabre*, a disease that affects the rich and the poor alike, in all the historical periods. Syphilis is referred in Cervante’s *Don Quixote* as “morbo gálico,” and tuberculosis plays a key role in a number of operas such as Verdi’s *La Traviata* and Puccini’s *La Bohème*. However, it is undoubtedly in cinematography that infectious diseases have been most often represented, not only in numbers but also in variety. Although there are >200 infectious diseases described in humans, along with around a hundred that impact animals or plants, not all of these diseases have been depicted in commercial movies. In the forthcoming section, the films selected portray the symptoms of the disease accurately, in addition to highlighting other aspects such as its social or historical impact or the medical treatment applied, with a view to use it as a teaching tool. Table 1 included a more detailed list of movies related to infectious diseases.

**Table 1.** Non-exhaustive list of movies and TV-series related to diseases caused by microbial pathogens that can be used to explain different scientific and humanistic aspects.

Disease	Title	Year	Director	Scientific and cultural aspects depicted	IMDB link
AIDS	120 BPM (*120 battements par minute*)	2017	Robin Campillo	AIDS, AZT, based on real events, HIV, Kaposi sarcoma, mycosis, patient care, public health, radiotherapy, safe-sex, social activism, social discrimination	https://www.imdb.com/title/tt6135348/
	And the Band Played On	1993	Roger Spottiswoode	AIDS, based on real events, contact tracing, Ebola outbreak, epidemiology, HIV, public health, social activism, social discrimination	https://www.imdb.com/title/tt0106273/
	Angels in America	2003	Mike Nichols	AIDS, AZT, based on real events, Kaposi sarcoma, patient care, social discrimination	https://www.imdb.com/title/tt0318997
	Dallas Buyers Club	2013	Jean-Marc Vallée	AIDS, AZT, based on real events, clinical assay, HIV, patient care, pseudoscience, social discrimination	https://www.imdb.com/title/tt0790636/
	Philadelphia	1993	Jonathan Demme	AIDS, HIV, Kaposi sarcoma, patient care, social discrimination	https://www.imdb.com/title/tt0107818/
	Savage Nights (*Les nuits fauves*)	1992	Cyril Collard	AIDS, biography, HIV, Kaposi sarcoma, radiotherapy, self-destructive behavior, toxic relationship, unsafe-sex	https://www.imdb.com/title/tt0105032/
Anthrax	The Hot Zone (second season)	2019	Brian Peterson, Kelly Shoulders, Jeff Vintar	Anthrax outbreak, based on real events, bioweapon, laboratory of microbiology	https://www.imdb.com/title/tt4131818/
	The Power of the Dog	2021	Jane Campion	Anthrax, bioweapon, cattle disease	https://www.imdb.com/title/tt10293406/
Cholera	Death in Venice (*Morte a Venezia*)	1971	Luchino Visconti	6th Cholera epidemic, concealment of the disease, public health measures	https://www.imdb.com/title/tt0067445/
	The Horseman on the Roof (*Le hussard sur le toit*)	1995	Jean-Paul Rappeneau	1832 Cholera epidemy, antiseptic procedures, quarantine, refugees	https://www.imdb.com/title/tt0113362/
	The Painted Veil	2006	John Curran	Cholera outbreak in China, disease symptoms, laboratory of microbiology, public health measures	https://www.imdb.com/title/tt0446755/
COVID-19	The Good Doctor (S4, E1 and E2)	2017	David Shore	COVID-19 outbreak, isolation protocol, lockdown, patient care, pre-pandemic situation, psychological effects on health care workers	https://www.imdb.com/title/tt6470478/
	Chicago Med (S6, E1 to E6)	2015	Michael Brandt, Derek Haas, Matt Olmstead	COVID-19 outbreak, isolation protocol, lockdown, patient care, psychological effects on health care workers	https://www.imdb.com/title/tt4655480/
Diphtheria	Balto	1995	Simon Wells	Based on real events, infantile diphtheria, anti-diphtheria serum	https://www.imdb.com/title/tt0112453/
	The Country Doctor	1909	D.W. Griffith	Infantile diphtheria in early 20th century USA	https://www.imdb.com/title/tt0000833/
	Togo	2019	Ericson Core	Based on real events, infantile diphtheria, anti-diphtheria serum	https://www.imdb.com/title/tt5116302/
Ebola	93 Days	2016	Steve Gukas	2014 Ebola outbreak in Lagos (Nigeria), based on real events, biocontainment, contact tracing, patient care, public Health	https://www.imdb.com/title/tt5305246/
	Outbreak	1995	Wolfgang Petersen	Antisera, biocontainment, bioweapons, contact tracing, disease expansion map, fictional Ebola-like virus outbreak, laboratory of microbiology, zoonosis	https://www.imdb.com/title/tt0114069/
	The Hot Zone (1st season)	2019	Brian Peterson, Kelly Shoulders, Jeff Vintar	Based on real events, biocontainment, Ebola outbreak, laboratory of microbiology	https://www.imdb.com/title/tt4131818/
Foot-and-mouth disease	Hud	1963	Martin Ritt	Animal culling, cattle disease, foot-and-mouth disease	https://www.imdb.com/title/tt0057163/
Fowl cholera	Birdman of Alcatraz	1962	John Frankenheimer	Animal disease, based on real events, pre-antibiotic era treatment of a disease	https://www.imdb.com/title/tt0055798/
Gangrene	Game of Thrones (S1-E9)	2011	David Benioff, D.B. Weiss	Gangrene, leprosy, wound infection	https://www.imdb.com/title/tt0944947/
	Gone with the Wind	1939	Victor Fleming	Gangrene, surgery, wound infection	https://www.imdb.com/title/tt0031381/
	The 12th Man (Den 12 Mann)	2017	Harald Zwart	Auto-surgery, based on real events, frostbite, gangrene	https://www.imdb.com/title/tt3300980/
	The Impossible	2012	J.A. Bayona	Antibiotics, based on real events, gangrene, wound infection,	https://www.imdb.com/title/tt1649419/
	The Profession of Arms (*Il mestiere delle armi*)	2001	Ermanno Olmi	Based on real events, gangrene, surgery, wound infection	https://www.imdb.com/title/tt0245276/
	The Road	2009	John Hillcoat	Gangrene, wound infection	https://www.imdb.com/title/tt0898367/
Flu	1918	1985	Ken Harrison	1918 flu pandemic in USA, based on real events	https://www.imdb.com/title/tt0088645/
	Flu (*Gamgi*)	2013	Sung-su Kim	Biocontainment, contact tracing, epidemic lockdown, H5N1 flu outbreak	https://www.imdb.com/title/tt2351310/
	Mission: Impossible II	2000	John Woo	Bioweapon, modified flu virus	https://www.imdb.com/title/tt0120755/
	Station Eleven	2021	Patrick Somerviller	Flu pandemic, lockdown, wipe out mankind	https://www.imdb.com/title/tt10574236/
	The Stand	1994	Mick Garris	Bioweapon, flu pandemic, wipe out mankind	https://www.imdb.com/title/tt0108941
Gonorrhea	*La vida alegre*	1987	Fernando Colomo	AIDS, gonorrhea, public health, prevention, syphilis	https://www.imdb.com/title/tt0092170/
Kuru	We are what we are	2013	Jim Mickle	Kuru, neurodegenerative disease, prion	https://www.imdb.com/title/tt2309021/
Leprosy	Ben-Hur	1959	William Wyler	Leprosy, leper colony	https://www.imdb.com/title/tt0052618/
	Braveheart	1995	Mel Gibson	Leprosy, medieval history	https://www.imdb.com/title/tt0112573/
	House of the Dragon (Season 1)	2022	Ryan J. Condal, George R.R. Martin	Fantasy, leprosy-like disease, patient care	https://www.imdb.com/title/tt11198330/
	Kingdom of Heaven	2005	Ridley Scott	Leprosy, medieval history, prosthetic face	https://www.imdb.com/title/tt0320661/
	Molokai	1999	Paul Cox	Based on real events, leprosy, leper colony, pre-antibiotic era treatment	https://www.imdb.com/title/tt0165196/
	*Molokai, la isla maldita*	1959	Luis Lucia	Based on real events, leprosy, leper colony	https://www.imdb.com/title/tt0053075/
	Papillon	1973	Franklin J. Schaffner	Based on real events, leprosy, leper colony	https://www.imdb.com/title/tt0070511/
	Sweet Bean (*An*)	2015	Naomi Kawase	Leper colony, social discrimination of the sick by law	https://www.imdb.com/title/tt4298958/
Malaria	Gold	2016	Stephen Gaghan	Based on real events, economy, geology, malaria seizure	https://www.imdb.com/title/tt1800302/
	The African Queen	1951	John Huston	Malaria seizure, mosquitoes	https://www.imdb.com/title/tt0043265/
Measles	Red Beard (*Akahige*)	1965	Akira Kurosawa	19th century Japan, bioethics, measles, syphilis	https://www.imdb.com/title/tt0058888/
	The Citadel	1938	King Vidor	Bioethics, early 20th century Britain, medical practice, measles outbreak, microbiology laboratory, silicosis, tuberculosis, typhoid fever outbreak	https://www.imdb.com/title/tt0029995/
Nipah	Virus	2019	Aashiq Abu	Based on real events, biological containment, contact tracing, epidemiology, nipah virus, outbreak, public health, zoonosis	https://www.imdb.com/title/tt8941440/
Plague	*I promessi sposi*	1922	Mario Bonnard	17th century plague epidemic in Italy, refugees	https://www.imdb.com/title/tt0196024/
	Flesh and Blood	1985	Paul Verhoeven	16th century plague, bioweapons, disease symptoms	https://www.imdb.com/title/tt0089153/
	*La Peste*	2018	Rafael Cobos, Alberto Rodríguez	16th century plague epidemic in Spain, ancient medicine	https://www.imdb.com/title/tt5868826/
	Nosferatu	1922	F.W. Murnau	Plague, syphilis, zoonosis,	https://www.imdb.com/title/tt0013442/
	Nosferatu the Vampyre	1979	Werner Herzog	Plague, zoonosis	https://www.imdb.com/title/tt0079641/
	Panic in the Streets	1950	Elia Kazan	Antibiotics, contact tracing, One Health, pneumonic plague, public health	https://www.imdb.com/title/tt0042832/
	*Pest in Florenz*	1919	Otto Rippert	14th century bubonic plague epidemic in Italy, E. A. Poe’s tale, lockdown	https://www.imdb.com/title/tt0010559/
	The Masque of the Red Death	1964	Roger Corman	14th century bubonic plague epidemic in Italy, E. A. Poe’s tale, lockdown	https://www.imdb.com/title/tt0058333/
	The Physician	2013	Philipp Stölzl	11th century bubonic plague epidemic in Persia, disease symptoms, epidemiology	https://www.imdb.com/title/tt2101473/
	The Plague (*La Peste*)	1992	Luis Puenzo	Bubonic plague outbreak in modern times, concealment of the disease, lockdown	https://www.imdb.com/title/tt0105127/
	The Seventh Seal (*Det sjunde inseglet*)	1957	Ingmar Bergman	Dance of death, depiction of bubonic plague in medieval times, disease symptoms	https://www.imdb.com/title/tt0050976/
	World without End	2012	Michael Caton-Jones	14th century bubonic plague epidemic in England, disease symptoms	https://www.imdb.com/title/tt1878805/
	Your Friend the Rat	2007	Jim Capobianco	14th century bubonic plague pandemic, insect vector, zoonosis	https://www.imdb.com/title/tt1134859/
Polio	Breathe	2017	Andy Serkis	Based on real events, mechanical respiration, polio	https://www.imdb.com/title/tt5716464/
Rabies	28 Days Later	2002	Danny Boyle	Bioweapons, eye infection, rabies virus	https://www.imdb.com/title/tt0289043/
	28 Weeks Later	2007	Juan Carlos Fresnadillo	Asymptomatic carrier, rabies virus	https://www.imdb.com/title/tt0463854/
	Cujo	1983	Lewis Teague	Animal diseases, rabies	https://www.imdb.com/title/tt0085382/
	The Crazies	1973	George A. Romero	Biological containment, rabies virus modified as a bioweapon, water contamination	https://www.imdb.com/title/tt0069895/
	The Crazies	2010	Breck Eisner	Biological containment, rabies virus modified as a bioweapon, Stevens-Johnson syndrome, water contamination	https://www.imdb.com/title/tt0455407/
Scarlett fever	Little Women	1949	Mervyn LeRoy	Disease sequels, scarlet fever in infants	https://www.imdb.com/title/tt0041594/
	The Reader	2008	Stephen Daldry	Scarlett fever in infants	https://www.imdb.com/title/tt0976051/
Smallpox	*22 Ángeles*	2016	Miguel Barden	19th century Europe smallpox epidemic, based on real events, Dr Balmis expedition, vaccination	https://www.imdb.com/title/tt5471634/
	80 000 Suspects	1963	Val Guest	Antiseptic procedures, autoclave sterilization, epidemiology, imported disease, smallpox outbreak, vaccination	https://www.imdb.com/title/tt0056802/
	A Royal Affair (*En kongelig affære*)	2012	Nikolaj Arcel	18th century Europe, based on real events, smallpox, variolization	https://www.imdb.com/title/tt1276419/
	Before Tomorrow (*Le jour avant le lendemain*)	2008	Marie-Hélène Cousineau Madeline Ivalu	Indigenous communities and imported diseases, smallpox	https://www.imdb.com/title/tt0929259
	The Killer that stalked New York	1950	Earl McEvoy	1947 smallpox outbreak USA, based on real events, disease expansion map, vaccination	https://www.imdb.com/title/tt0042643/
	The King’s Whore (*La putain du Roi*)	1990	Axel Corti	17th century Europe, based on real events, smallpox	https://www.imdb.com/title/tt0100440/
	*Variola Vera*	1982	Goran Markovic	1971 smallpox outbreak Europe, based on real events, biocontainment, imported disease, vaccination	https://www.imdb.com/title/tt0083275/
Syphilis	Dangerous Liaisons	1988	Stephen Frears	18th century Europe, syphilis	https://www.imdb.com/title/tt0094947/
	Miss Evers’ Boys	1997	Joseph Sargent	Based on real events, bioethics, human experimentation, syphilis, Tuskegee experiment	https://www.imdb.com/title/tt0119679/
	Out of Africa	1985	Sydney Pollack	Based on real events, cerebral malaria, Salvarsan, syphilis	https://www.imdb.com/title/tt0089755/
	The English	2022	Hugo Blick	Congenital syphilis, disease symptoms, syphilis	https://www.imdb.com/title/tt11771270/
	The Libertine	2004	Laurence Dunmore	17th century Europe, based on real events, disease symptoms, syphilis	https://www.imdb.com/title/tt0375920/
	The Quiet Duel (*Shizukanaru kettô*)	1949	Akira Kurosawa	Congenital syphilis, social stigma, syphilis	https://www.imdb.com/title/tt0041870/
	VD Plan Attack	1973	Les Clark	Gonorrhea, syphilis	https://www.imdb.com/title/tt0399832/
Tetanus	The Children of Huang Shi	2008	Roger Spottiswoode	Based on real events, cholera, malaria, typhus, tetanus, wound infection	https://www.imdb.com/title/tt0889588/
Typhoid fever	The Knick	2014	Steven Soderbergh	Asymptomatic carrier, infantile meningitis, plague, public health, syphilis, tuberculosis, typhoid fever outbreak	https://www.imdb.com/title/tt2937900/
Tuberculosis	*Kameliadamen*	1907	Viggo Larsen	Tuberculosis	https://www.imdb.com/title/tt0000583/
	Camille	1936	George Cukor	Tuberculosis	https://www.imdb.com/title/tt0028683/
	Charité (Season 1)	2017	Sabine Thor-Wiedemann, Jakob Hein, Christine Otto.	Based on real events, diphtheria, microbiology laboratory, tuberculosis	https://www.imdb.com/title/tt5337806/
	City of Joy	1992	Roland Joffé	Leprosy, leper colony, medical volunteer, tuberculosis	https://www.imdb.com/title/tt0103976/
	Moulin Rouge!	2001	Baz Luhrmann	Disease symptoms, tuberculosis	https://www.imdb.com/title/tt0203009/
	My Neighbor Totoro (*Tonari no Totoro*)	1988	Hayao Miyazaki	Sanatorium, tuberculosis	https://www.imdb.com/title/tt0096283/
	The Magic Mountain (*Der Zauberberg*)	1982	Hans W. Geissendörfer	Philosophy, sanatorium, tuberculosis	https://www.imdb.com/title/tt0084946/
	The Wind Rises (*Kaze Tachinu*)	2013	Hayao Miyazaki	Based on real events, tuberculosis	https://www.imdb.com/title/tt2013293/
	Tombstone	2003	George P. Cosmatos, Kevin Jarre	Based on real events, tuberculosis	https://www.imdb.com/title/tt0108358/
Yellow fever	Yellow Jack	1938	George B. Seitz	Based on real events, clinical assay, human experimentation, insect vector, yellow fever	https://www.imdb.com/title/tt0030990/

It should be noted that some movies can be placed in more than one category. In the case of some TV series, the season (S) and episodes (E) are indicated, if needed.

### AIDS

#### Philadelphia

##### Director: Jonathan Demme (1993)

In 1993, Jonathan Demme’s film *Philadelphia* marked a turning point in the way society viewed and treated people with AIDS (Aijón 2005). At that time, antiretroviral therapy was in its early stages and only managed to delay the inevitable, so the viewer becomes a witness to the gradual physical deterioration of the character played by Tom Hanks. However Demme shows the decay in a way that is neither lurid nor sensationalist, but with great care and respect, as exemplified by the trial sequence, in which the effects of the Kaposi’s sarcoma on Hank’s body are seen as a reflex in a mirror. At the same time, the lawyer played by Denzel Washington undergoes a moral transformation, culminating in the famous library sequence, where he becomes aware of what it means to be discriminated against not by race, but by a disease. *Philadelphia* helped change the society’s view of people with AIDS and opened the door for more movies to address STDs. In fact, a search for “AIDS” in the keywords section of IMDB yields >600 titles, with the earliest one from 1985. In contrast, a search for “tuberculosis” yields little >200 titles, with the earliest from 1910.

### Anthrax

#### The Power of the Dog

##### Director: Jane Campion (2021)

Early in the film, a group of cowboys is seen driving cattle toward a village, and in the distance, the bloated carcass of a cow is visible. Their leader, Phil (Benedict Cumberbatch), warns the other cowboys to keep the cattle away, explaining that the cow died from anthrax, a disease caused by *Bacillus anthracis*. Campion uses the “Chekhov’s gun” dramatic resource to introduce this animal disease in the plot. Later on, a feud develops between Phil and his nephew Peter (Kodi Smit-McPhee). Peter, who is studying medicine, harbors a unique plan to kill his uncle. He found the carcass of a cow that has died of anthrax, so it contains spores of the bacteria. With his gloved hands and the help of a scalpel, he takes part of the hides. The occasion arises when Phil is plating a lasso with rawhide and Peter notices that he has a wound on his hand. So he passes him the hides of the infected animal. The next day, Phil wakes up feverish, dizzy, and with swollen, blackened hand. A doctor is called but nothing can be done to save him.

### Cholera

#### The Painted Veil

##### Director: John Curran (2006)

The *Painted Veil* is a 1925 novel written by William Somerset Maugham, which has been adapted for the cinema on several occasions, this last one being the most faithful to the original story. Edward Norton plays a British doctor in early 20th-century China fighting a cholera outbreak in a place where medical resources are limited and drastic public health measures should be implemented (Cross 2007, Han and Curtis 2020). A particularly impactful sequence in the film takes us on a visit to a rural hospital, revealing the harsh reality of confronting such a devastating disease. The camera captures that the afflicted individuals lie on a plank with a central hole for the evacuation of the “rice-water” diarrhea. The film also outlines the mechanisms of disease transmission through contaminated water and illustrates the public health interventions taken to address the outbreak. An interesting aspect is the exploration of the tension between cultural traditions and the need for preventive measures. This clash is typically resolved through prompt and pragmatic actions.

### COVID-19

#### The Good Doctor

##### Creator: David Shore (2017)

Different movies and television series were the first to reflect the Covid-19 pandemic (Cambra-Badii 2022, Pappas 2023). Interestingly, the start of *The Good Doctor*’s fourth season showed the initial moments of the outbreak, when the symptoms of the disease were still not very well defined and many patients were misdiagnosed with flu. After that we see the ravages of the epidemic, the countless problems due to the shortage of resources, the development of the new hospital protocols to manage the disease, and of course the numerous death of patients and also the psychological burden caused on the medical staff.

### Diphtheria

#### Togo

##### Director: Ericson Core (2019)

In January 1925, Dr Curtis Welch reported that there was an outbreak of infantile diphtheria in the Alaskan town of Nome, with 20 confirmed cases and another 50 suspected cases. Weather conditions prevented the shipment of anti-diphtheria serum by air, so it was decided that an attempt would be made to bring in the serum using a relay race on dog sleds. A nine-kilogram package containing 300 000 units (about 500 doses) of anti-diphtheria serum would be taken from Seward to the town of Nenana by rail, and from there relays would carry it to Nome. The distance between Nenana and Nome is 1085 km and was covered by several teams in only 5 days and 7 h through a blizzard that reached −31°C. The feat was incredible because under normal conditions it would take 15 days to complete. A total of 150 dogs and 20 “mushers” who drove the sleds took part. Although the death of five children was regretted, 300 000 units that arrived in Nome made it possible to control the outbreak until the rest of the anti-diphtheria serum arrived in mid-February.

### Ebola

#### 93 Days

##### Director: Steve Gukas (2016)

On 25 March 2014, the World Health Organization (WHO) reported an Ebola virus outbreak in Guinea, impacting multiple populations and raising concerns about its potential spread to other African nations. By May, the virus had reached Conakry, and in July, Freetown became affected. On 20 July 2014, an infected Liberian diplomat arrived in Lagos, Nigeria. Through the dedicated efforts of the healthcare team at the First Consultant Medical Center, the outbreak was successfully contained, preventing its dissemination in a city with a population of 20 million (Shuaib et al. 2014). The central figure in this narrative is Dr Ameyo Adadevoh, portrayed by actress Bimbo Akintola. Dr Adadevoh’s critical role lay in her early suspicion that the patient’s symptoms indicated Ebola. She promptly ordered his isolation pending test results, even at the risk of potential diplomatic tensions. Concurrently, she instructed the medical staff on implementing preventive measures, despite the inadequate availability of personal protective equipment (PPE) for such a situation. The Nigerian government alerted the WHO and imposed the confinement of all healthcare workers who had interacted with the infected individual. While popular belief often associates Ebola with extensive hemorrhaging, the film sheds light on the fact that many victims succumb to organ failure, particularly of the kidneys or liver. All individuals who had contact with the healthcare workers were closely monitored, with a requirement to undergo a 21-day quarantine. Ultimately, Nigeria recorded 20 cases of Ebola, resulting in 8 fatalities, including several among those who had treated the infected Liberian diplomat, including Dr Adadevoh herself.

### Foot-and-mouth disease

#### Hud

##### Director: Martin Ritt (1963)

Paul Newman plays Hud, a quarrelsome and egocentric cattle rancher. His cattle contract foot-and-mouth disease and are quarantined pending test results. Upon confirmation, all the cattle must be culled. The cattle-slaughter scene is very famous. It was supervised by the Humane Society, so no animal was hurt.

Patricia Neal won the Oscar for her melancholic performance. It was due to a personal tragedy as she had tragically lost her daughter to encephalitis caused by measles. Her husband at that time was Roahl Dahl, who in 1986 wrote a letter detailing this personal tragedy while encouraging people to get vaccinated (Dahl 1986).

### Flu

#### The Stand

##### Director: Mick Garris (1994)

The most devastating pandemic ever suffered by mankind was the influenza of 1918–1920. It is estimated that there were 500 million cases and caused between 17 and 100 million deaths (Taubenberger 2006). Stephen King was inspired by this pandemic to write his novel *The Stand*. Published in its definitive form in 1990, it was not long before being adapted into a 6-h television series. In *The Stand*, a flu virus is genetically engineered to serve as a bioweapon. Named Captain Trips, this virus wreaks havoc by wiping out a 99.99% of humanity. Its transmission occurs both through fomites and through airborne particles, rendering conventional protective measures and vaccination ineffective. Within 2 weeks, the virus spreads globally, leading to the collapse of social and healthcare systems. Deaths are not solely attributable to the disease itself but are exacerbated by the resulting societal breakdown. The few remaining survivors will try to rebuild civilization.

While the notion of an ultimate biological weapon that kills everyone might appear clichéd, the underlying truth may be more unsettling. At the height of the Cold War, many scientists wondered about the probability of self-destruction and determined that there were two critical parameters: the number of people capable of destroying the planet and the likelihood that they will do so. The use of atomic bombs was limited by their complexity and cost, yet biotechnology’s accessibility and affordability have changed this (Sotos 2019). While biotechnology holds the potential to address diseases, it also harbors the capability to produce new biological weapons with no known remedies.

### Gangrene

#### Il mestiere delle armi (The Profession of Arms)

##### Director: Ermanno Olmi (2001)

Gangrene appears in countless war-themed films. From the stench of mature cheese as in *Saving Private Ryan*, or a surgeon amputating an infected limb from a poor wretch pleading not to lose it as shown in *Gone with the Wind*. There are times when medical intervention can save the patient as happens with Naomi Watts in *The Impossible*. However, more often than not, cinema depicts the dire fate of those who suffer from wound infections, such as Khal Drogo’s character in the *Game of Thrones*. The beautifully crafted Italian movie *Il mestiere delle armi* tells the story of the final days of the famous *condottiero* Giovanni de’ Medici, who fought during the 16th century Italian wars. In 1526, he suffered a leg injury from a cannon shot. He was treated by his personal physician, the Jew Abramo Arié, who applied the knowledge of the time, such as washing the wound with ointments and applying leeches to eliminate the inflammation (Vesalius’ anatomy was still 17 years away). They also placed roses on the headboard to give fragrance and disguise the stench. As the infection worsened, a decision was made on the third day to amputate the leg. Interestingly, we saw how Abramo heats the knives and saws over a flame, presumably to quickly cauterize the wound. According to witnesses, Giovanni de Medici himself refused to be immobilized and in the absence of adequate lighting, he held with his own hands the candelabra that illuminated the operation. However, the infection was already widespread and Giovanni passed away two days later.

### Gonorrhea

#### La vida alegre (The Joyful Life)

##### Director: Fernando Colomo (1987)

The story revolves around a young female doctor who oversees a welfare center situated in the heart of a Madrid slum. The center’s primary aim is to provide advice and treatment to prostitutes and drug addicts regarding sexually transmitted diseases. Meanwhile, her husband, an ambitious and rising politician, views the center as a political advantage for his career. However, the doctor feels a sense of duty to safeguard public health and must navigate not only the distrust of those she’s assisting but also the numerous political hurdles. Additionally, she must confront a marital crisis triggered by her husband’s promiscuity. With such a plot, one would expect a social drama, but instead we find a screwball comedy. From a microbiological perspective, the film offers a brief overview of various sexually transmitted diseases, including gonorrhea, syphilis, chlamydia, AIDS, and more. It also illustrates the chain of contagion and the repercussions of disregarding precautions when engaging in promiscuous behavior. Interestingly, despite its release in 1987, the film barely touches on the topic of AIDS, a disease considered at that time as a “silent epidemic” marked by stigma.

### Leprosy

#### Molokai: The story of Father Damien

##### Director: Paul Cox (1999)

Based on the biography of the Father Damien written by Hilde Eynikel, this film was shot on the island of Molokai, where leprosy patients who were interned there still reside today. In fact, many of them acted in the film. Father Damien arrived on the island in 1873, the same year that Gerhard Hansen described leprosy as being caused by a bacterium now known as *Mycobacterium leprae*. At that time, it was thought that leprosy was a form of syphilis. The movie vividly portrays the appalling conditions of the lazaretto where all the lepers were confined. The film also depicts the progression of the disease of Father Damien until his death. While the treatment of leprosy with dapsone was established in the 1950s and later improved with the antibiotics clofazimine and rifampicin, Molokai’s isolation laws remained in effect until 1969. Despite being cured, the internees decided to continue living on Molokai out of fear of being stigmatized due to the scars left by the disease.

### Malaria

#### The *African Queen*

##### Director: John Huston (1951)

Malaria is a common theme in movies set in the jungle, such as *The Bridge on the River Kwai, The Wages of Fear, Mountains of the Moon*, etc. In C. S. Forester’s novel, *The African Queen*, Rose and Charlie are affected by malaria due to the bites of mosquitoes during the crossing of the Bora River Delta, and describe its conditions as follow. *Every morning they were prostrated by it, almost simultaneously. Their heads ached, and they felt a dull coldness creeping over them, and their teeth began to chatter, until they were helpless in the paroxysm, their faces drawn and lined and their finger-nails blue with cold*. They survive by treated themselves with quinine. In this John Huston’s masterpiece, the fever only struck Charlie and Rose comforts him. The shooting of the film was made in Africa and during the production, Bogart and Huston were the only members of the cast and crew who escaped illness. They attributed their immunity to consuming whiskey on location instead of the local water.

### Measles

#### The Citadel

##### Director: King Vidor (1938)

The Citadel is the most famous work of the physician and writer A.C. Cronin. It recounts his personal experiences as a doctor serving the miners of a Welsh village and even summarized his findings on the relationship between silicosis and tuberculosis. The main character is Dr Manson, an altruistic idealist dedicated body and soul to improve the sanitary conditions. Shortly after its publication, it was adapted by King Vidor, albeit with fundamental plot alterations. The book not only touches upon tuberculosis but also deal with two other microbiological topics. One is the expeditious way in which Dr Manson stops an outbreak of typhoid fevers caused by the leakage of fecal water into the drinking supply. The other pertains to childhood measles. In a specific scene, Manson visits a house to examine a child recovering from measles and discovers that his brother, although asymptomatic, has attended school. He promptly heads to the school, urging the child’s return home to prevent an outbreak. However, the teacher refuses as it would mean forfeiting the free glass of milk provided to the children.

### Nipah virus

#### Virus

##### Director: Aashiq Abu (2019)

In May 2018, an outbreak of the Nipah virus occurred in the Indian state of Kerala (Ajith Kumar and Anoop Kumar 2018). The outbreak impacted 19 individuals and initiated when a patient was brought to the emergency department at the University Hospital in the city of Kozhikode. This patient exhibited symptoms including febrile illness, shortness of breath, vomiting, and convulsions. Initially, the condition was suspected to be dengue or rabies, but a subsequent molecular diagnosis confirmed Nipah virus infection. Following this confirmation, strict usage of PPE became standard practice, alongside quarantine measures and contact tracing efforts. A series of meetings with policymakers ensued, aiming to determine whether the outbreak’s origin was natural or if there was a potential connection to intentional bioterrorist activities. Alongside this investigation, public health measures were implemented to address the situation. Efforts were made to inform the public and mitigate panic, with a particular emphasis on advising against consuming fruit that might have been exposed to bites from fruit bats, which are the natural reservoir of the virus. In light of contact tracing, a quarantine involving 2000 people was implemented across the Kozhikode and Malappuram districts. While some patients were administered the monoclonal antibody M 102.4 as part of their treatment, only two patients were able to recover, while the others succumbed to the virus. The outbreak was officially declared to be over on 10 June 2018.

### Plague

#### La Peste (The Plague)

##### Creators: Rafael Cobos and Alberto Rodríguez (2018)

The plague outbreaks that swept across Europe from the 14th to the 17th century, have been extensively treated in the literature, and so, also in the movies. Plague was an “egalitarian” disease that struck both the rich and the poor, throughout different historical periods. One of the most notable examples is Edgar Allan Poe’s *The Mask of the Red Death: A Fantasy*, which has been adapted on several occasions, with *Die Pest in Florenz* (1919) and *The Masque of the Red Death* (1966) being the more famous adaptations. Other noteworthy representations about the plague in past times are *I promessi sposi* (1922), *The Sevent Seal* (1957), *Flesh and Blood* (1985), *World Without End* (2012), and *The Physician* (2013). Many of these films consistently include anachronisms, and one of the most famous examples can be found in *The Seventh Seal*, set in 13th century Sweden, even though the Black Death did not reach Europe until the mid-14th century. The Spanish TV series *La Peste* also features similar errors, along with an overuse of clichés such as the evil inquisitor. However, it is worth watching due to its quality and historical backdrop. The series transports us to Seville in 1597, although in reality, the actual outbreak occurred in 1649, resulting in the deaths of 60 000 people (45% of its population). One of the most haunting and somber scenes unfolds within the confines of an unsanitary hospital, where a “plague doctor” navigates among patients. While the sequence is undeniably eerie, it is historically inaccurate, as this costume was actually invented by the Frenchman Charles de Lorme in 1620. Another mistake pertains to the character of physician Nicolas Monardes, who lived between 1493 and 1588 and was a pioneering figure in botany. He was renowned across Europe for his work “*Historia medicinal de las cosas que se traen de nuestras Indias Occidentales*,” with its first volume dating back to 1560. Contrary to the series portrayal, he was never prosecuted by the Spanish Inquisition.

### Polio

#### Breathe

##### Director: Andy Serkis (2017)

This biopic tells the history of Robert Cavendish, who contracted polio at age 28. The disease paralyzed him from the neck down, so he needed a mechanical respirator to breathe, making him a “responaut.” Initially, he was depressed by his condition, but fortunately his quality of life was improved, thanks to the help of his wife Diane, family, and friends. One of the improvements was the design and construction of a wheelchair with a built-in respirator that allowed Robert to travel away from home. He starts to campaign for the improvement of the quality of life of disabled people.

### Rabies

#### Cujo

##### Director: Lewis Teague (1983)

Bats are a natural reservoir for the rabies virus. In 2021, there were five cases of human rabies in the USA due to bat bites. Typically, this occurs when people try to catch bats, prompting defensive reactions. In this film, an unvaccinated St. Bernard dog contracts rabies after being bitten by a bat, becoming a killer. The movie follows genre norms with tense scenes, calculated scares, violent deaths, and a climactic showdown. During production, six different dogs, including a Rottweiler, were used, along with a robotic head and disguised human for certain shots. Interestingly, the dogs enjoyed the filming, wagging their tails, so they had to tie their tails to their bodies. To film the scene of the car attack, a simple trick was employed: hiding dog toys within the vehicle and instructing the dog to “Fetch!”. The foam and thick saliva around the dog’s mouth was made using egg white and sugar, a treat for the dogs.

### Scarlet fever

#### Little Women

##### Director: Mervyn LeRoy (1949)

Louisa May Alcott’s famous semi-autobiographical novel condenses her experiences over several years during the American Civil War and part of the post-war era. According to several literary critics, Louisa May Alcott’s is considered one of the first feminist writers, as she portraits how a young woman, like the character Jo, strives to establish herself as a writer and attain economic independence. *Little Women* has been adapted to film numerous times, with the 1933, 1949, and 1994 versions being the most well known. One of the most famous parts involves the character Beth falling ill with scarlet fever after assisting a German immigrant family in need of help with their children. In those times, it was understood that surviving scarlet fever granted immunity, and thus, Meg and Jo take on the task of caring for her. Little Amy, however, is sent to her aunt’s house to avoid contagion. Though Beth initially overcomes the crisis, her health declines over the course of 2 years until she ultimately passes away. Louisa May Alcott drew inspiration from her own sister Elizabeth’s experience. On her deathbed, she asked for ether to ease her pains. However, in the film adaptation, Beth’s death is portrayed in a more softened manner as it seems that she simply sleeps after having a conversation with her sister.

### Smallpox

#### Before Tomorrow

##### Directors: Marie-Hélène Cousineau, Madeline Ivalu (2008)

This is the first film made by a collective of Inuit women, all the actors are Inuit and, of course, it is filmed in the Inuit language with accompanying subtitles. The story is set circa 1840 somewhere in the Arctic Circle. Amid the summer season, a pair of Inuit clans convene for trade, fishing to amass provisions for the impending winter, and jubilantly commemorating a union in marriage. During the evenings, they share tales and gossip. Among their discussions is the revelation that they have encountered individuals from afar who arrived in a “big canoe without paddles.” These strangers engaged in commercial transactions, yet unbeknownst to them, they also introduced smallpox. The theme of how an imported disease can wipe out an indigenous population appears in other films (e.g. *Medicine Man*, 1992). The originality of this film lies in its internal perspective, recounting the events from within the community itself. The character of the grandmother vividly conveys the enormity of the loss, not only of human lives but also of all the traditions and culture that these clans represented.

### Syphilis

#### The English

##### Creator: Hugo Blick (2022)

Until the end of the 20th century, STDs were rarely mentioned in films. Even a director like John Huston shied away from referencing Toulouse-Lautrec’s syphilis in his 1952 film *Moulin Rouge*. Only the master Kurosawa showed in *The Quiet Duel* the shame and stigma suffered by those who suffer from a disease that generates a deep social rejection. After the AIDS epidemic in the 1980s, the taboo on STDs began to be lifted and the gruesome burdens of those diseases were represented on the screen without hesitation. Some examples are *Miss Evers’ Boys, Dangerous Liaisons, The Libertine*, and *The Knick*. This recent TV-series depicts very vividly the symptoms and disfigurement caused by *Treponema pallidum* infection in the secondary and tertiary phases of the syphilis, and also the effects of congenital syphilis.

### Tetanus

#### The Children of Huang Shi

##### Director: Roger Spottiswoode (2008)

This melodrama is based on the real-life account of journalist George Hogg. In 1938, he arrived in Shanghai and embarked on humanitarian endeavors amidst the backdrop of the Sino–Japanese war. By 1944, he was in charge of an orphanage in Huang Shi, accommodating 60 children. Faced with the mandatory conscription from the Chinese Army, Hogg orchestrated a daring escape to Lanzhou, traveling a 700-km journey on foot through mountainous and snow-covered terrain. From there they were taken by truck to the city of Shandan where they established the new orphanage. A few months later, while playing a basketball game with his pupils, Hogg cut his toe. The wound became infected and he began to develop tetanus. Desperate to secure tetanus serum, two of the boys embarked on a 500-km motorcycle journey, but by the time they returned, Hogg had died. In the movie, the screenwriters opted for a more dramatic portrayal. Here, Hogg sustains a wound on his hand amidst the desert, grappling with a sand tornado while endeavoring to repair one of the trucks.

### Typhoid fever

#### The Knick

##### Director: Steven Soderbergh (2014)

We now live in a time of endless possibility. More has been learned about the treatment of the human body in the last 5 years than was learned in the previous 500. Twenty years ago, 39 was the number of years a man could expect from his life. Today, it is more than 47. This speech is featured in the first episode of a TV-series created by Jack Amiel and Michael Begler. Their inspiration was drawn from the history of Knickerbocker Hospital in 1900s’ New York—a world marked by backwardness and inequality, yet on the verge of rapid transformation through technology and science. Electricity is the latest technological advance, surgical techniques are being developed, aseptic procedures such as the use of carbolic nebulizers, have just been implemented and infectious diseases are one of the main causes of mortality. One of the plots is the control of the outbreak of typhoid fever that was caused by Mary Mallon, better known as “Typhoid Mary” (Marineli et al. 2013). Other infectious diseases such as tuberculosis, syphilis, infantile meningitis, plague, septicemia, etc. also appear. The series had the supervision of Dr Stanley Burns, founder of the Burns Archive of Historical Medical Photographs. Although it is fiction, some of its protagonists are based on historical characters. For example, Clive Owen gives life to Dr John W. Thackery, who is a transcript of Dr William Stewart Halsted, the famous surgeon who founded John Hopkins Hospital and who, apart from developing the surgical technique of radical mastectomy, was also addicted to cocaine and morphine.

### Tuberculosis

#### Moulin Rouge!

##### Director: Baz Luhrmann (2001)

If there is a love triangle characteristic of the 19th century, it is the one formed by the young bourgeois, the pretty girl and Mycobacterium tuberculosis (Day 2017). This romantic trio appeared for the first time in the play *The Lady of the Camellias* by Alexandre Dumas published in 1848. It was later adapted to opera by Verdi (*La Traviata*), but the bohemian touch was given by Puccini in his work *La Boheme*. Logically, the story has also been made into a film on numerous occasions, “*Moulin Rouge!*” being one of the most recent. Although one already knows how the story is going to end, the truth is that the film manages to catch you, either by its aesthetics, its performers, or its soundtrack. In the course of the film, we see how the beautiful Satine (Nicole Kidman) is slowly wasting away due to tuberculosis. The symptoms are clear: pallor, cough, hemoptysis, fainting, and above all, dyspnea. Although the name of the disease is never mentioned, her beloved Cristian (Ewan McGregor) describes it in the following words: *a force darker than jealousy and stronger than love had begun to take hold of Satine*.

## Scientists

It is paradoxical that cinematography is an art that exists, thanks to scientific and technological progress, and at the same time the relationship between cinema and science is not easy. A scientist tries to understand the universe, a filmmaker tries to express his vision of the universe. One might expect that the character of scientists in movies would always be treated under a positive light, but that has not been the case, even from their origins. The archetype of the “mad scientist” capable of destroying the world was born in 1927 with Fritz Lang’s *Metropolis*. Something similar happens with the character of the microbiologist. Initially, the pioneers of microbiology knowing as the “microbe hunters,” were solitary heroes. They even fight the diseases in the harsh conditions of developing countries. However, little by little, the character changed and the “mad microbiologist,” capable of destroying life on the Earth, thanks to biological weapons, also appeared. At the same time, it was evident that science advances were made by teams of scientist, not by solitary figures. In the 1953 classic *The War of the Worlds*, a multidisciplinary group of researchers are assembled in order to cope with an alien invasion. If we consider that an infectious disease outbreak is one of the worst menaces, it is not surprise that the best way to deal with that is to gather a group of heroic epidemiologists, as we can see in movies like *Outbreak* and *Contagion* (Lynteris 2016). This section discusses six films that best represent the work of microbiologists and show the evolution from the “lone hero” to the “multidisciplinary team.” Table 2 lists other movies that are related to scientist work.

**Table 2.** Non-exhaustive list of movies and TV-series related to microbial and epidemiological scientists.

Title	Year	Director	Microbial aspect depicted	Real scientists depicted	IMDB link
*22 ángeles*	2016	Miguel Barden	19th century Europe, based on real events, Dr Balmis expedition, smallpox, vaccination	Francisco Balmis, José Salvany, Isabel Zendal Gómez	https://www.imdb.com/title/tt5471634/
80 000 Suspects	1963	Val Guest	Antiseptic procedures, autoclave sterilization, contact tracing, epidemiology, imported disease, smallpox outbreak, vaccination		https://www.imdb.com/title/tt0056802/
And the Band Played On	1993	Roger Spottiswoode	AIDS, based on real events, contact tracing, Ebola outbreak, epidemiology, HIV, public health, social activism, social discrimination	Donald Francis, Robert Gallo, James Curran, Luc Montaigner, Françoise Barre, Selma Dritz	https://www.imdb.com/title/tt0106273/
Arrowsmith	1931	John Ford	Anthrax, Bubonic plague, laboratory research, peer pressure, phage therapy		https://www.imdb.com/title/tt0021622/
Birdman of Alcatraz	1962	John Frankenheimer	Animal disease, based on real events, Fowl cholera, pre-antibiotic era treatment of a disease	Robert Stroud	https://www.imdb.com/title/tt0055798/
*Casas de fuego*	1995	Juan Bautista Stagnaro	Based on real events, Chagas disease, public health	Salvador Mazza	https://www.imdb.com/title/tt0109385/
Charité (Season 1)	2017	Sabine Thor-Wiedemann, Jakob Hein, Christine Otto.	Based on real events, diphtheria, microbiology laboratory, tuberculosis	Robert Koch, Emil Behring, Paul Ehrlich, Rudolf Virchow	https://www.imdb.com/title/tt5337806/
Contagion	2011	Steven Soderbergh	Based on SARS 2002 outbreak, biocontainment, contact tracing, laboratory of microbiology, lockdown, pandemic, R0, vaccine, zoonosis		https://www.imdb.com/title/tt1598778/
Doctor Bull	1933	John Ford	Typhoid fever outbreak		https://www.imdb.com/title/tt0023955/
Dr. Ehrlich's Magic Bullet	1940	William Dieterle	Antibiotic, based on real events, microbiology laboratory, salvarsan, syphilis	Robert Koch, Emil Behring, Paul Ehrlich, Sahachiro Hata	https://www.imdb.com/title/tt0032413/
Outbreak	1995	Wolfgang Petersen	Antisera, biocontainment, bioweapons, disease expansion map, fictional Ebola-like virus outbreak, laboratory of microbiology, zoonosis		https://www.imdb.com/title/tt0114069/
Panic in the Streets	1950	Elia Kazan	Antibiotics, One Health, pneumonic plague, public health		https://www.imdb.com/title/tt0042832/
Robert Koch: The Battle Against Death (*Robert Koch, der Bekämpfer des Todes*)	1939	Hans Steinhoff	Anthrax, based on real events, germ theory of disease, experiments, microbiology laboratory, vaccines, tuberculosis	Robert Koch, Rudolf Virchow, Georg Gaffky, Friedrich Löffler	https://www.imdb.com/title/tt0031868/
The Andromeda Strain	1971	Robert Wise	Alien pathogen, biological containment, laboratory of microbiology, sterilization		https://www.imdb.com/title/tt0066769/
The Citadel	1938	King Vidor	Bioethics, early 20th century Britain, medical practice, measles outbreak, silicosis, tuberculosis, typhoid fever outbreak.		https://www.imdb.com/title/tt0029995/
The Hot Zone (1 and 2 seasons)	2019	Brian Peterson, Kelly Shoulders, Jeff Vintar	Anthrax outbreak, based on real events, bioterrorism, biocontainment, Ebola outbreak, laboratory of microbiology	Nancy Jaax	https://www.imdb.com/title/tt4131818/
The Nun’s Story	1959	Fred Zinnemann	Based on real events, laboratory of microbiology, leprosy, malaria, tuberculosis	Marie Louise Habets	https://www.imdb.com/title/tt0053131/
The Story of Louis Pasteur	1936	William Dieterle	Anthrax, based on real events, laboratory of microbiology, rabies, vaccines	Louis Pasteur, Emile Roux, Joseph Lister	https://www.imdb.com/title/tt0028313/
Virus	2019	Aashiq Abu	Based on real events, biological containment, epidemiology, Nipah virus, outbreak, public health, zoonosis		https://www.imdb.com/title/tt8941440/
Yellow Jack	1938	George B. Seitz	Based on real events, clinical assay, human experimentation, insect vector, yellow fever	Walter Read, Carlos Finlay, Aristides Agramonte, James Carroll William Gorgas, Jesse W. Lazear	https://www.imdb.com/title/tt0030990/

### Arrowsmith

#### Director: John Ford (1931)

In the 1930s, the emergence of sound films coincided with the research and development of the first substances with antimicrobial properties. At that time, thanks to Paul de Kruif’s *Microbe Hunters*, infectious diseases were seen as a threat that science could defeat, and so, several biopics dedicated to the fathers of microbiology such as *The Story of Louis Pasteur, Robert Koch: The Battle Against Death*, and *Dr. Ehrlich’s Magic Bullet* were made (Kirby 2013). However, the distinction of having the first microbiologist portrayed on the big screen goes to a fictional character. The best-selling novel *Arrowsmith*, written by Sinclair Lewis, was adapted for the screen by John Ford in 1931. It achieved significant acclaim from both critics and audiences, receiving four Oscar nominations, including the Best Picture. The film addresses various issues pertinent to scientific vocation, such as the pressure to make groundbreaking discoveries and publish scientific papers (the well-known “publish or perish” mentality). It also deals into the disillusionment that comes with discovering that other researchers have preceded you in your work, the challenges of securing funding for research, the prevalence of sensationalized science in the media, ethical dilemmas surrounding clinical trials involving human subjects, and the personal sacrifices one makes in the pursuit of scientific passion (García-Sánchez and García-Sánchez 2005). Naturally, microbiology plays a prominent role throughout the film. Arrowsmith grapples with infantile diphtheria, embarks on the development of a serum for symptomatic anthrax caused by *Clostridium chauvoei*, engages in the exploration of bacteriophages, and experiences disappointment upon realizing that Felix d’Herelle has beaten him to the discovery. The film’s “final apotheosis” centers around his battle against an outbreak of bubonic plague on a Caribbean island, alongside his efforts to design a clinical trial to assess the effectiveness of his phage therapy. While it may appear somewhat naive and antiquated by today’s standards, there are many aspects in which this movie remains relevant. In fact, it effectively captures the essence of pursuing a scientific career as a means of livelihood.

### The Nun’s Story

#### Director: Fred Zinnemann (1959)

The “missionary scientist” film stereotype could be defined as a medical doctor or a scientist who goes to a developing country to combat infectious diseases. There are several examples, such as the characters played by Rock Hudson in *The Spiral Road* and Patrick Swayze in the *City of Joy*. However, one of the best is the Sister Luke played by Audrey Hepburn in *The Nun’s Story*. In 1956, writer Kathryn Hulme published a novel based on the biography of her friend Marie Louise Habets. Director Fred Zinnemann acquired the rights to adapt it into a film and cast Audrey Hepburn in the lead role. Hepburn dedicated time to immerse herself in various convents and master the use of a microscope in order to bring the character of Sister Luke to life. She also had the opportunity to meet and befriend Marie Louise Habets. Notably, Audrey Hepburn’s portrayal of Sister Luke is very likely to be the first female microbiologist ever played on film. Much of the narrative is set in the former Belgian Congo, where Sister Luke aspires to carry out her missionary work. However, before she can fulfill her mission, she must undergo training as a nurse at the Institute of Tropical Medicine in Antwerp. During this phase, we observe Sister Luke learning to perform mycobacterial stains to identify and distinguish between Koch’s bacillus and Hansen’s bacillus. At one point, she imparts to a classmate that they are very alike. Both rod-shaped, both acid-fast, both with a slight shadow almost like an enclosing capsule. However, if you look very closely you will see that the leprosy bacillus is slightly fatter and longer, a factual distinction (5 microns versus 8). In another scene, Sister Luke undergoes an oral parasitology examination where she is questioned about the trypanosome causing sleeping sickness and the clinical manifestations of malaria. Subsequently, her journey takes her to a leper colony situated in the jungle. This particular sequence was filmed in an authentic lazaretto, providing a glimpse of real patients grappling with the lingering effects of the disease.

### The Andromeda Strain

#### Director: Robert Wise (1971)

*The Andromeda Strain* is the film adaptation of Michael Crichton’s 1969 novel of the same name. Directed by Robert Wise, this techno-thriller follows a team of scientists as they work to identify and control an extraterrestrial pathogenic microorganism that induces severe blood clotting in its victims. Notably, the laboratory tests and equipment represented were state-of-the-art in 1971. These included the recreation of a high-level biosafety lab, culturing the microorganism in different media, sizing Andromeda by passing contaminated air through a series of filters with increasing pore size, preparing and observing samples under an electron microscope, and capturing an X-ray diffraction image of an Andromeda crystal (Sánchez 2011b). Interestingly, the film became embroiled in a controversy related to animal welfare, particularly concerning a scene in which a monkey is exposed to Andromeda and falls death. In truth, no animals were harmed during filming, as Wise ensured all scenes adhered to ASPCA guidelines. For the notable sequence, the monkey was placed in a box with breathable air, inside a room filled with carbon dioxide. An assistant with and autonomous respiratory equipment and with an additional mask for the monkey was placed off camera. When the mechanical arm opened the box, the monkey was briefly exposed to CO_2_, causing it to faint. Wise continued filming for a couple of seconds before the assistant revived the monkey by placing the mask on. The scene was achieved in a single take.

### And the Band Played On

#### Director: Roger Spottiswoode (1993)

This TV docudrama, based on journalist Randy Shilts’ book, describes the early stages of the epidemiological battle against AIDS and the scientific controversies surrounding the discovery of HIV. It starts in 1981 at the CDC in Atlanta, where Don Francis and Jim Curran initiate an investigation into an unusual outbreak of diseases affecting homosexuals in San Francisco and Los Angeles, caused by opportunistic pathogens like *Pneumocystis carinii* (then mistaken for a protozoan). Through diligent detective work, they determine that the mysterious disease is caused by a sexually and blood-transmitted virus. The film also portrays the heated dispute between Luc Montagnier and Robert Gallo’s groups over who was the first to discover the virus. Despite its merits, the film contains notable flaws due to its creation before a clear understanding of the disease’s origin emerged. One major issue is the concept of “patient zero” and the notion that AIDS could have been curtailed in 1982 with increased CDC funding (Sharp 2011). In hindsight, we now know this notion is misguided, as the disease actually originated in Africa in the early 20th century and emerged in 1981 due to its impact on the first-world population. While additional funding might have reduced casualties and enabled earlier prevention campaigns, it would not have altered the course of the disease’s global spread.

### Outbreak

#### Director: Wolfgang Petersen (1995)

This film so full of errors that make it amusing to dissect. Let us start with the first major biological mistake: capuchin monkeys are not found in Africa; they inhabit the jungles of Central America. Despite its flaws, the film has its merits. It opens with a haunting phrase from Nobel laureate Joshua Lederberg: *The single biggest threat to humanity’s continued dominance on the planet is the virus*. The plot is engaging and deals with various epidemiological issues. One such theme is the emergence of new diseases from previously inaccessible parts of the world. It also explores the spread of zoonoses through illegal animal trafficking. Additionally, it raises concerns about the development of biological weapons that can evolve beyond control and lack an antidote. The film interestingly outlines the scientists’ discussions on transmission chains, methods of virus spread, and quarantine measures to halt contagion. Particularly notable is the scene in the movie theater where one of the infected coughs and generates an aerosol formed by droplets full of virus. Through a zoom, we see how these droplets spread to the other spectators. The Motaba virus is a typical “movie virus” that kills in <48 h with 100% mortality and causes horrible symptoms to anyone infected. Similar to *Panic in the Streets*, researchers must race against time to find a remedy and prevent the pathogen’s spread. Calculating the antibody concentration of an antiviral serum for 2700 people obtained from the blood of a single capuchin monkey, is a challenge that I leave to the reader.

### Contagion

#### Director: Steven Soderbergh (2011)

It is likely that this is one of the most-watched movies during lockdown. Released in 2011, it passed through screens without much attention. However, it has become famous after the COVID-19 pandemic. Screenwriter Scott Z. Burns and director Steven Soderbergh decided from the outset that it had to be realistic and not sensational. They sought advice from Lawrence Brilliant and Ian Lipkin and drew from the SARS epidemic of 2002–4 to recreate a possible scenario and design the MEV-1 virus as a fusion of two paramyxoviruses: the Nipah virus and a porcine rubulavirus. The idea was for the virus to have high lethality like Nipah, and at the same time be highly contagious like the rubulavirus that causes mumps. Soderbergh crafted this film in a style similar to what he did previously in *Traffic* and the drug world. He intertwines a series of parallel stories whose common thread is the epidemic. We encounter regular people, doctors striving to combat the epidemic, scientists attempting to figure out how to develop a vaccine, politicians making decisions that will impact millions of people, and even charlatans trying to exploit others’ suffering. It is truly astonishing how much this 2011 film foreshadowed various situations we have recently experienced: lockdowns, hoarding of supplies, social distancing, and the race to find a vaccine. In contrast to other movies where crises are resolved within days, here it is depicted that the scientific process is slow, painstaking, and meticulous, spanning months. This progress relies on both government support and pharmaceutical companies. Two key aspects of this film have been studied with more detail. The first one is the public distrust on the actions taken by the public health officials such as quarantines and lockdowns (Han and Curtis 2020). The second is the paradox that the pro-vaccination and pro-science tone of the movie could have create an over-simplification of a complex problem and even encourage vaccine hesitancy during the actual SARS-CoV2 pandemic (McGuire 2021).

## Microbial dystopias

### Biotechnological fears

Mary Shelley’s “Frankenstein” and H.G. Wells’ “The Island of Doctor Moureau” are the origin of a plot of many science fiction movies. A scientist manipulates biology to create something that at first seems beneficial but then has unintended consequences. Knowledge about pathogenic microorganisms allows us to develop more effective therapies against them, but it also allows the development of biological weapons. At other times, however, evil occurs unintentionally. For example, the development of an experimental therapy causes the emergence of an epidemic that wipes out humanity. There are several films with this anti-biotechnology message, warning us that the alteration of the natural order of things, including diseases, can lead to catastrophic outcomes. The blockbusters *I Am Legend* and *Rise of the Planet of the Apes* are the best examples of that. Fortunately, this cautionary message has not deterred advancements in anti-tumor therapies based on oncolytic viruses (Verma 2013, Srivastava and Riddell 2015). Table 3 lists some of the movies related to these categories.

**Table 3.** Non-exhaustive list of movies and TV-series related to microbial dystopias.

Category	Title	Year	Director	Microbial aspect depicted	IMDB link
Bioweapons	Inferno	2016	Ron Howard	Bacterial growth, Bartlett’s Beaker, biocontainment	https://www.imdb.com/title/tt3062096/
	Mission: Impossible II	2000	John Woo	Modified flu virus that infects red blood cells	https://www.imdb.com/title/tt0120755/
	Rampage	2018	Brad Peyton	CRISPR, genetic modification	https://www.imdb.com/title/tt2231461/
	The Cassandra Crossing	1976	George P. Cosmatos	Biocontainment, modified plague bacteria	https://www.imdb.com/title/tt0074292/
	The Crazies	1973	George A. Romero	Biological containment, rabies virus modified as a bioweapon, water contamination	https://www.imdb.com/title/tt0069895/
	The Crazies	2010	Breck Eisner	Biological containment, rabies virus modified as a bioweapon, Stevens-Johnson syndrome, water contamination	https://www.imdb.com/title/tt0455407/
	The Hot Zone (second season)	2019	Brian Peterson, Kelly Shoulders, Jeff Vintar	Anthrax outbreak, based on real events, bioterrorism, laboratory of microbiology	https://www.imdb.com/title/tt4131818/
	The Last Man on Earth	1964	Ubaldo Ragona, Sidney Salkow	Microbiology laboratory, pandemic, vampires, wipe out mankind	https://www.imdb.com/title/tt0058700/
	The Patriot	1998	Deam Semler	Antiviral therapy, modified virus	https://www.imdb.com/title/tt0120786/
	The Power of the Dog	2021	Jane Campion	Anthrax, cattle disease	https://www.imdb.com/title/tt10293406/
	The Omega Man	1971	Boris Sagal	Pandemic, wipe out mankind	https://www.imdb.com/title/tt0067525/
	The Satan Bug	1965	John Sturges	Biocontainment, Botulinum toxin laboratory of microbiology, modified polio virus	https://www.imdb.com/title/tt0059678/
	Warning Sign	1985	Hal Barwood	Biocontainment, genetic engineering, laboratory of microbiology, modified bornavirus, transgenic bacteria, zombification	https://www.imdb.com/title/tt0090293/
Biotechnological application goes awry.	I am Legend	2007	Francis Lawrence	Animal experimentation, biocontainment, genetic engineering, laboratory of microbiology, viral vector therapy, zombification virus	https://www.imdb.com/title/tt0480249/
	Resident Evil: The Final Chapter	2016	Paul W.S. Anderson	Biocontainment, biotech company, bioweapons, progeria, genetic engineering, viral vector therapy, wipe-out mankind, zombification virus	https://www.imdb.com/title/tt2592614/
	Rise of the Planet of the Apes	2011	Rupert Wyatt	Animal experimentation, Alzheimer disease, disease expansion map, genetic engineering, pandemic, viral vector therapy	https://www.imdb.com/title/tt1318514/
	The Bourne Legacy	2012	Tony Gilroy	Genetic engineering on humans, viral vector therapy	https://www.imdb.com/title/tt1194173/
Microbial apocalypse	12 Monkeys	1995	Terry Gilliam	Bioweapons, pandemic, wipe out mankind	https://www.imdb.com/title/tt0114746/
	Carriers	2009	David Pastor, Àlex Pastor	Antiseptic procedures, bioethics, pandemic, wipe out mankind	https://www.imdb.com/title/tt0806203/
	No Blade of Grass	1970	Cornel Wilde	Ecological disaster, plant virus, world famine	https://www.imdb.com/title/tt0066154/
	The Stand	1994	Mick Garris	Bioweapons, flu pandemic, wipe out mankind	https://www.imdb.com/title/tt0108941
Zombie apocalypse	28 Days Later	2002	Danny Boyle	Animal experimentation, bioweapons, eye infection, modified rabies virus	https://www.imdb.com/title/tt0289043/
	28 Weeks Later	2007	Juan Carlos Fresnadillo	Asymptomatic carrier, modified rabies virus	https://www.imdb.com/title/tt0463854/
	In the Flesh	2013	Dominic Mitchell	Anti-zombie therapy, social discrimination, zombification	https://www.imdb.com/title/tt2480514/
	Infection (*Infección*)	2019	Flavio Pedota	Political protest, zombification virus	https://www.imdb.com/title/tt6022266/
	Maggie	2015	Henry Hobson	Patient care, progressive infection, zombification virus	https://www.imdb.com/title/tt1881002/
	REC	2007	Jaume Balagueró, Paco Plaza	Biocontainment, lockdown, zombification virus	https://www.imdb.com/title/tt1038988/
	REC 4: Apocalypse	2014	Jaume Balagueró	Annelid vector, zombification virus	https://www.imdb.com/title/tt1649443/
	Resident Evil	2002	Paul W.S. Anderson	Bioweapons, biocontainment, biotech company, zombification virus	https://www.imdb.com/title/tt0120804/
	The Girl with All the Gifts	2016	Colm McCarthy	Pandemic, fungal pathogen, fungal zombification, symbiosis, wipe-out mankind	https://www.imdb.com/title/tt4547056/
	The Last of Us	2023	Neil Druckmann, Craig Mazin	Pandemic, fungal pathogen, fungal zombification, symbiosis, wipe-out mankind	https://www.imdb.com/title/tt3581920/
	The Returned	2013	Manuel Carballo	Anti-zombie therapy, biotechnology, social discrimination, zombification	https://www.imdb.com/title/tt2093270/
	The Walking Dead	2010–2022	Frank Darabont	Endogenous virus, pandemic, wipe out mankind, zombification virus	https://www.imdb.com/title/tt1520211/
	World War Z	2013	Marc Forster	Biocontainment, epidemiology, pandemic, zombification virus	https://www.imdb.com/title/tt0816711/

#### I Am Legend

##### Director: Danny Boyle (2002)

Richard Matheson’s novel *I am Legend* published in 1954 describes a post-apocalyptic world that has been devastated after a nuclear war that triggers a mosquito-borne zoonosis, in which a strain of *Bacillus* has wiped out 99% of humanity and transformed almost all the survivors into vampire creatures. Robert Neville is the sole survivor and devotes his efforts to understanding the disease and trying to develop a cure. Matheson’s book has several readings, from the anguish of human loneliness and the displacement from one cultural form to another. The novel has been adapted into a film four times, the most recent being this one starring Will Smith. In this version, a genetically modified measles virus intended as a cancer therapy triggers the pandemic that transforms humans into sentient zombie-like creatures.

#### Rise of the Planet of the Apes

##### Director: Rupert Wyatt (2011)

In 1968, the science fiction masterpiece titled *Planet of the Apes* was released, leaving a lasting impact. The film’s success led to the creation of four sequels and even a television series, although none could quite match the brilliance of the original. Charlton Heston’s iconic image before the ruins of the Statue of Liberty became a symbol of that era. Drawing inspiration from the third film of the saga, *Conquest of the Planet of the Apes*, Fox decided to rejuvenate the ape saga. The resulting movie, *Rise of the Planet of the Apes* fared well and marked the beginning of a new film series. This time, a biotech company develops a groundbreaking therapy for Alzheimer’s disease. By modifying a retrovirus to stimulate brain tissue neurogenesis, they enhance cognitive abilities in apes during testing. Although the therapy initially improves cognitive functions in afflicted humans, their immune systems eventually counter the virus, negating its effects. As expected, the narrative intertwines the avarice of large biotech corporations with military conspiracies. A new therapeutic virus, modeled after the flu virus, is engineered to be highly aggressive and inhaled for maximum impact. While this virus significantly enhances ape cognitive abilities, it comes with unintended consequences: it spreads like the flu and results in human fatality within 48 h. When an infected human, particularly an airline pilot responsible for transcontinental flights, triggers an outbreak, the consequences are catastrophic. The image accompanying the end credits aptly illustrates how diseases can spread via air travel.

### Zombie apocalypse

There is no doubt that the actual zombie icon is a creation of George A. Romero. Chronologically, *The night of the living dead* (1968) was not the first zombie movie, but undoubtedly is the cast of all current ones, since according to the IMDB there are 4330 titles (and rising) of movies, TV series, or video games, in which zombies appear in one way or another. Some examples are the aforementioned *World War Z*, the *Resident Evil Saga*, or the TV series *The Walking Dead* and *The Last of Us*. Usually, in those movies, mankind has been brought to the brink of extinction due to the action of a zombifying virus. For this last section, three titles have been chosen for comment as the most interesting from a microbiological perspective, but the reader can find some other movies in Table 3.

The truth is that zombies are important players in the science of epidemiology. “If you’re prepared for a zombie apocalypse, you’re prepared for a tornado, an earthquake, or other catastrophes” seems to be the motto that the United States Center for Disease Control (CDC 2023) uses to aware people that they should have a kit in their homes containing the essentials items to face any kind of eventuality. Likewise, the zombie pandemic is considered the “worst case” scenario for infectious diseases, so mathematical models have been developed to try to understand how it would spread and how to face such a situation efficiently. Spoiler alert, the scientific conclusion is the one advanced by Max Brooks in his book *World War Z*: the only way for humanity to survive is to quarantine and eliminate all those infected (Munz et al. 2009, Lofgren 2016).

#### 28 Days Later

##### Director: Danny Boyle (2002)

Unlike the vast majority of zombie movies, *28 Days Later* portrays the infected as incurably ill individuals who have contracted a virus. Their symptomatology is clear, and they can die from lack of food or fatal organ injuries, rather than being immortal putrefied beings that do not die unless their brains are destroyed. The film is a modern adaptation of the 1962 British sci-fi movie *The Day of the Triffids*, replacing carnivorous plants with zombies. Here, animal rights activists target a military facility, inadvertently releasing a mutant form of the rabies virus known as the Rage virus. This highly contagious and virulent virus spreads through saliva and blood, entering the body through mucous membranes, including the eye’s conjunctiva, as illustrated in a striking sequence. Within a minute of infection, a person becomes a frenzied maniac with bloodshot eyes, attacking only those who remain uninfected. The film achieved significant success, followed by its sequel *28 Weeks Later*, directed by Juan Carlos Fresnadillo. In that one, the Rage virus epidemic has been brought under control by a simple method, all infected people in the British Isles have simply been left to starve to death. After a deemed safe quarantine period, repopulation occurs. However, new inhabitants encounter a survivor who happens to be an asymptomatic carrier, setting the stage for what unfolds next. Beyond the gory elements, these two productions offer scenarios that can be used to explain and discuss various scientific topics. For instance, pathogen virulence, strategies for pathogen spread in a population, animal experimentation, challenges arising from laboratory raids by activists, the concept of incubation time, and more.

#### The Walking Dead

##### Creator: Frank Darabont (2010)

In 2003, the comic book *The Walking Dead* (*TWD*) was published, created by writer Robert Kirkman and illustrator Tony Moore, who contributed to the first six issues. From the seventh issue onward, illustrator Charlie Adlard took over the series. The comic was quite successful and received praise from Max Brooks, the author of the best-seller *World War Z*. In 2010, AMC adapted the comic into a six-episode TV series directed by Frank Darabont, turning it into a cultural phenomenon. The show even inspired university courses that utilized its episodes to illustrate concepts in disciplines such as epidemiology, sociology, psychology, and history. The core idea of the series is that humans can be more dangerous than zombies in their pursuit of survival. *TWD* has left a lasting impact, with the last comic issue, the 193rd, published in July 2019. The TV series reached chapter 177, which aired on 20 November 2022. The *TWD* universe continues through spin-offs such as *Fear the Walking Dead* and *The Walking Dead: World Beyond*, suggesting zombies will remain prominent for some time. Microbiology plays a pivotal role in two instances within the story. At the end of the first season, the protagonists arrive at the CDC in Atlanta, encountering virologist Edwin Jenner, who studies the virus that turns humans into zombies. Scientific accuracy is not well maintained in this episode, particularly in the way Jenner handles his experiments. A comparison with the careful, realistic handling of dangerous samples in *The Andromeda Strain* underscores the discrepancies in episode *TS-19*. The virologist in *TWD* processes tissue samples carelessly and manages to view the virus’s triple nucleic acids under an optical microscope lacking a magnifying lens. However, the notion that zombification is caused by a virus present in human DNA, activated upon death, is intriguing. This concept is based on the fact that 10% of our genome originates from retroviruses inserted millions of years ago. Some of these retroviral genes encode syncytins, essential for fetal implantation in the placenta (Mi et al. 2000). Microbiology reappears in the fourth season, where a swine flu outbreak nearly eradicates the main characters. Drawing inspiration from the 1918 flu pandemic and Albert Camus’ novel *The Plague*, the screenwriters convey the paranoia and claustrophobic atmosphere of those episodes.

#### The Last of Us

##### Creators: Neil Druckmann, Craig Mazin (2023)

In 2013, the video game *The Last of Us* was released. It falls under the category of “third-person” games, where players control a character navigating a zombie-infested world to complete a mission. Unlike other video games and movies, zombification in this game is caused not by a virus, but by a mutant fungus of the species *Ophiocordyceps unilateralis*. This fungus has crossed the species barrier to infect humans. The fungus is neurotropic, primarily targeting the host’s brain (Seppälä et al. 2008). Unlike traditional decomposing zombies, the infected bodies in this game undergo a transformation due to the invasion of fungal hyphae. The video game gained such fame that HBO recently decided to adapt it into a TV series. The introduction of the first episode is set in 1968, featuring a television interview with two scientists discussing the threat of pandemics to humans. They express concerns about viruses such as influenza, which can spread rapidly through air routes. While they believe our immune system is equipped to handle such threats due to co-evolution with those microbial pathogens over time, one scientist expresses less confidence about fungal pathogens. Fungi can manipulate a host’s mind through the production of psychoactive substances. Although he dismisses fungi as a threat due to their inability to survive human body temperature, he notes that an increase in temperature could prompt fungal adaptation, potentially posing a danger. While this sequence sets the stage effectively, it contains two significant inaccuracies. Firstly, pathogenic fungi that withstand human body temperature such as *Candida, Cryptococcus*, and *Aspergillus*, already exist. Secondly, much more important, evolution does not reason, it is a random process.

## Conclusions

There is a great diversity of microbial themes represented in popular movies that can be used as educational tools not only for life science students but also for other disciplines such as arts or history studies. Although in most featured films, microbes are the harbingers of disease and desperation, there are a few films that show that they can do useful things, even being the saviors of mankind. Also, the precise work of microbiologists usually is depicted correctly, especially in modern movies. Nevertheless, it is important that educators should be able to identify and correct the misconceptions that can be found in some films, and use them as an additional resource. It seems that these small creatures will continue to inspire screenwriters in the future.
